# Case Report: longitudinal immunoglobulin abnormalities preceding IgA-λ MGUS and colonic DLBCL: a case suggesting immune-driven B-cell dysregulation

**DOI:** 10.3389/fonc.2026.1878363

**Published:** 2026-08-12

**Authors:** Huifeng Qian, Min Dong, Guanqiao Shen

**Affiliations:** Shaoxing Second Hospital, Shaoxing, Zhejiang, China

**Keywords:** B-cell malignancy, diffuse large B-cell lymphoma, MGUS, monoclonal gammopathy, primary colonic lymphoma

## Abstract

Monoclonal gammopathy of undetermined significance (MGUS) is a common premalignant condition typically associated with progression to plasma cell disorders. Research suggests that MGUS may also coexist with or precede other lymphoid malignancies, potentially reflecting shared mechanisms involving chronic immune dysregulation. We report a 73-year-old man with more than 10 years of long-standing polyclonal immunoglobulin elevation, who subsequently developed IgA-λ MGUS and was later diagnosed with primary colonic diffuse large B-cell lymphoma (DLBCL). Molecular analysis identified mutations in *TET2*, *IDH2*, *IGLL5*, and *DUSP2*. Rather than indicating a direct clonal transformation, this case may reflect a multistep process involving chronic immune dysregulation and independent or parallel B-cell clonal events. These findings highlight the potential clinical significance of long-standing immunoglobulin abnormalities and suggest the potential value of longitudinal immune and molecular monitoring.

## 1 Introduction

DLBCL is the most common aggressive subtype of non-Hodgkin lymphoma, accounting for approximately 30% of all cases (1). The gastrointestinal tract is the most frequent extranodal site of involvement, with the stomach being the predominant location, whereas primary colonic lymphoma remains relatively uncommon (2). Due to the lack of specific clinical manifestations, such cases are often diagnosed incidentally during endoscopic evaluation. MGUS is a common premalignant plasma cell disorder characterized by the presence of a monoclonal immunoglobulin without evidence of symptomatic plasma cell malignancy (3). It is traditionally considered a precursor to multiple myeloma and related plasma cell disorders, with an average annual progression risk of approximately 1% (4).

Increasing evidence suggests that MGUS may also be associated with a broader spectrum of lymphoid malignancies beyond plasma cell disorders (5, 6). However, this association does not necessarily imply a direct clonal relationship. Instead, MGUS and other B-cell neoplasms may arise in the context of shared biological backgrounds, including chronic antigenic stimulation, immune dysregulation, and age-related hematopoietic changes (7, 8). In this setting, long-standing polyclonal immunoglobulin abnormalities may represent a manifestation of chronic immune dysregulation in selected individuals; however, their clinical significance, particularly regarding their potential association with subsequent MGUS and lymphoma development, remains unclear.

Here, we report a case of primary colonic DLBCL occurring in a patient with more than 10 years of long-standing immunoglobulin abnormalities, followed by the subsequent emergence of IgA-λ MGUS. We highlight a rare temporal sequence that raises the possibility of a shared immune-dysregulated background underlying both conditions.

## 2 Case presentation

### 2.1 Clinical information

A 73-year-old man with a 30-year history of hypertension presented to our hospital on January 2, 2026, after multiple colonic polyps were detected during routine colonoscopic screening performed under intravenous sedation. The patient had a long history of smoking (approximately 30 cigarettes per day) and reported no alcohol consumption. The specific antihypertensive medications used were unknown. Upon admission, the patient’s vital signs were stable: body temperature 36.6 °C, pulse rate 60 beats/min, respiratory rate 20 breaths/min, and blood pressure 109/68 mmHg. The patient was conscious and in generally good condition. No superficial lymphadenopathy was detected on physical examination.

Ten years earlier, during hospitalization for thyroid nodule surgery, laboratory tests revealed mildly elevated serum immunoglobulin levels, with IgA of 4.82 g/L (reference range 0.7-4.0 g/L) and IgM of 3.11 g/L (reference range 0.3-2.2 g/L). The thyroid-stimulating hormone (TSH) level was 0.063 mIU/L (reference range: 0.600-4.900 mIU/L), thyroglobulin antibody (TgAb) was 86.43 IU/mL (reference range: 0-4.0 IU/mL), and thyroid peroxidase antibody (TPOAb) was 0.01 IU/mL (reference range: 0-9.0 IU/mL). Longitudinal thyroid function parameters, including FT3, FT4, and TSH levels, are summarized in **Table 1**. Three years prior to admission, the patient was diagnosed with diabetes mellitus and received treatment with metformin and acarbose.

**Table 1:** The patient’s thyroid function index test results during the follow-up period.

Project	Reference	2015	2019	2021	2022	2023	2024	2026
FT3	4.00-6.10 pmol/L	4.98	5.64	5.27	5.49	4.83	5.63	5.42
FT4	8.50-14.50 pmol/L	13.21	11.68	12.77	13.71	12.65	16.07	15.08
TSH	0.600-4.900 mIU/L	0.063	0.873	1.328	1.239	0.827	0.276	1.734

**Figure 1:**
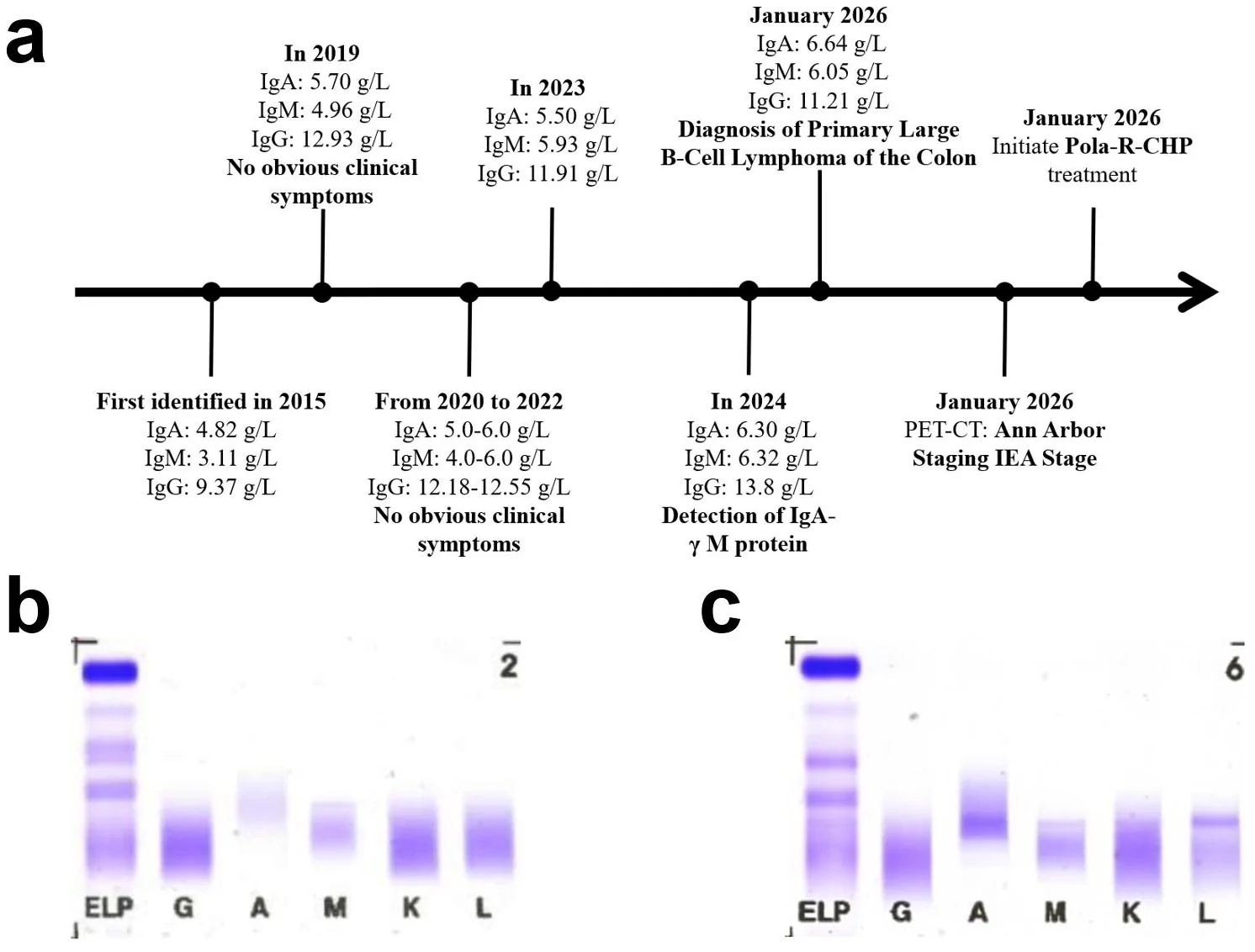
Timeline of the clinical course and evolution of immunological findings. **(A)** Serial serum immunoglobulin levels from 2015 to 2026. **(B)** Serum IFE pattern in 2021 showing no evidence of monoclonal gammopathy. **(C)** Serum IFE pattern in 2024 demonstrating the emergence of an IgA-λ monoclonal protein.

In 2021, serum immunofixation electrophoresis (IFE) showed no evidence of monoclonal gammopathy (**Figure 1B**). However, repeat testing in 2024 demonstrated the emergence of an IgA-λ monoclonal protein (**Figure 1C**). Quantitative immunoglobulin analysis showed elevated serum IgA (6.3 g/L) and IgM (6.32 g/L). The quantitative value of the monoclonal IgA component was not available. Serum LDH was 221 U/L (reference range: 0–248 U/L) (**Figure 1**). Serum free light chain analysis revealed a κ level of 30.23 mg/L (reference range: 3.3-19.4 mg/L) and a λ level of 42.81 mg/L (reference range: 5.7-26.3 mg/L), with a κ/λ ratio of 0.71. Peripheral blood morphology was normal. At that time, the patient declined bone marrow examination due to advanced age.

### 2.2 Laboratory and diagnostic findings after admission

#### 2.2.1 Laboratory findings

Serum free light chain analysis showed a kappa level of 32.26 mg/L (reference range 3.3-19.4 mg/L) and a lambda level of 59.11 mg/L (reference range 5.7-26.3 mg/L). Liver function tests revealed alanine aminotransferase (ALT) of 94 U/L (reference range 9–50 U/L) and aspartate aminotransferase (AST) of 65 U/L (reference range 15–40 U/L). LDH was 234 U/L (reference range 0–248 U/L). Serum potassium was 3.29 mmol/L (reference range 3.50-5.30 mmol/L). Bone marrow immunophenotypic analysis by flow cytometry showed that plasma cells accounted for 0.2% of nucleated cells, among which CD138-positive monoclonal abnormal plasma cells constituted 0.06% of nucleated cell counts and represented 30.0% of total plasma cells. The TSH was 1.734 mIU/L, thyroglobulin was 0 ng/mL (reference range: 1.59-50.30 ng/mL), TgAb was 122.3 IU/mL, TPOAb was 0.30 IU/mL, and IgG4 was 1.147 g/L (reference range: ≦1.400 g/L). It should be noted that the patient has been taking Levothyroxine (Euthyrox) long-term. The patient’s antinuclear antibody panel test result was negative. No other significant abnormalities were observed in the remaining laboratory parameters.

#### 2.2.2 Pathological findings

**Figure 2:**
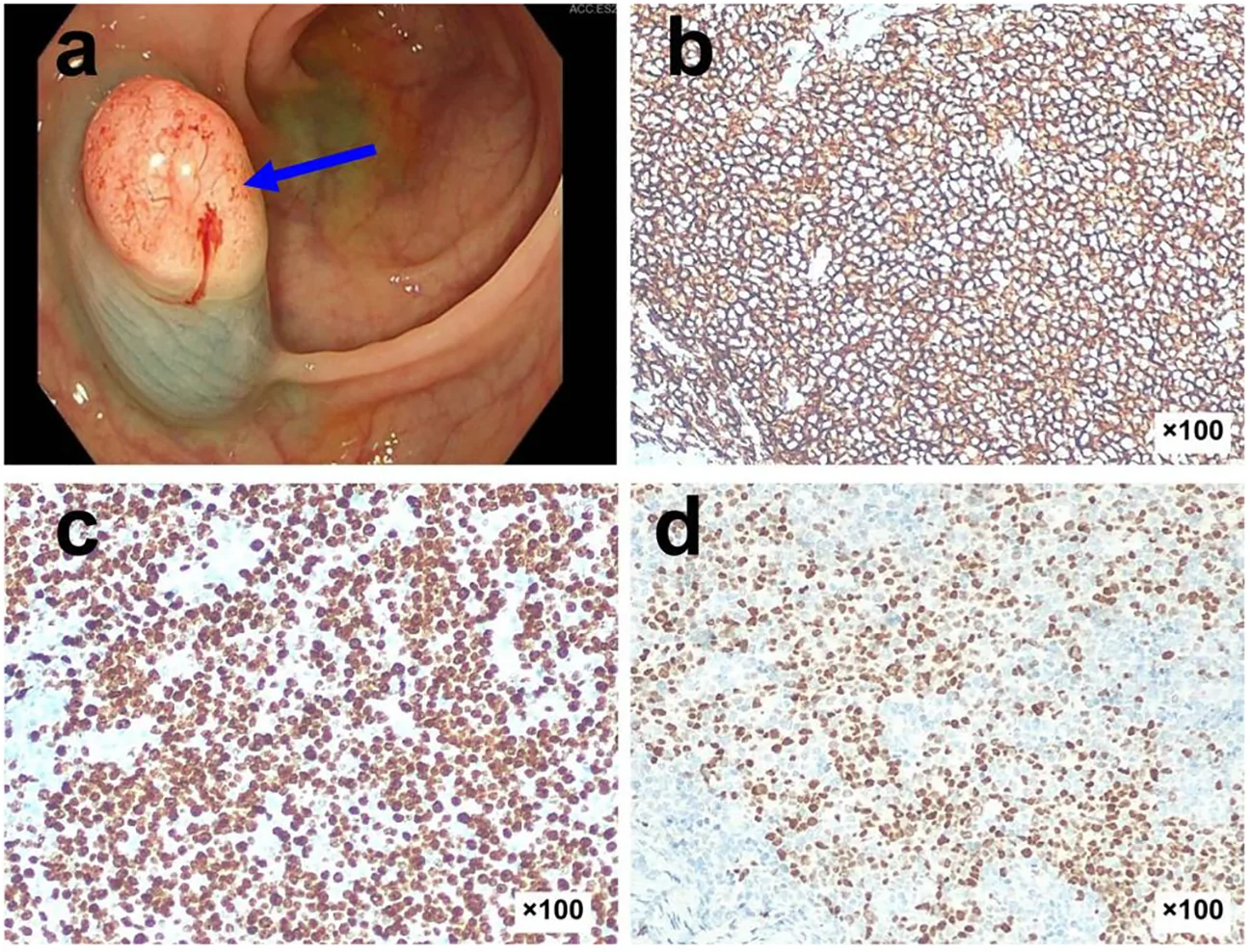
Colonoscopic and pathological findings: **(A)** Polypoid lesions; Immunohistochemistry: **(B)** CD20, **(C)** Ki-67, **(D)** Bcl-6.

During painless colonoscopy, multiple polypoid lesions were identified in the sigmoid colon, including a representative lesion measuring approximately 1.0 × 0.8 × 0.6 cm that was selected for biopsy (**Figure 2A**). Immunohistochemical analysis of the tumor showed positive staining for CD20 (**Figure 2B**), Ki-67 (90%) (**Figure 2C**), Bcl-6 (70%) (**Figure 2D**), and CD10, while EBER *in situ* hybridization was negative. Immunohistochemical staining for κ and λ light chains showed only weak and scattered positivity (<5%), and clear light chain restriction was not demonstrated. Based on these findings, the lesion was diagnosed as primary colonic diffuse large B-cell lymphoma.

#### 2.2.3 Next-generation sequencing findings

Targeted next-generation sequencing (NGS) was performed using a commercial hybridization-based capture panel covering 196 lymphoma-associated genes. The assay was designed to detect coding-region single nucleotide variants (SNVs) and small insertions/deletions (indels), with variants reported at a variant allele frequency (VAF) threshold of ≥1%.

Tumor tissue sequencing identified several genetic alterations, including IDH2: c.51G>A (p.R172K) (35.44%), DUSP2: c.401G>A (p.G134D) (2.22%), IGLL5: c.72G>A (p.W24X) (1.00%), IGLL5: c.175_177del (p. S59del) (5.17%), TET2: c.4210C>T (p.R1404X) (34.33%), and TET2: c.5591T>A (p.V1864E) (36.80%).

#### 2.2.4 Imaging findings

Positron emission tomography-computed tomography (PET-CT) demonstrated increased FDG uptake in the sigmoid colon, with a maximum standardized uptake value (SUVmax) of approximately 6.5. The disease was classified as Ann Arbor stage IEA. Multiple lymph nodes showed mild uptake but were small and low metabolic activity, and were therefore interpreted as reactive rather than definitive lymphoma involvement.

#### 2.2.5 Diagnostic considerations

The differential diagnosis included secondary gastrointestinal involvement by systemic lymphoma, extranodal marginal zone lymphoma, and plasmacytic neoplasms. However, the localized colonic lesion, absence of systemic lymphadenopathy or bone destruction, immunophenotype positive for CD20/CD10/Bcl-6, high Ki-67 index, and lack of plasma cell predominance supported the diagnosis of primary colonic DLBCL rather than plasma cell neoplasm or indolent lymphoma.

Regarding the monoclonal gammopathy, the patient did not show evidence of progression from MGUS to plasma cell myeloma and did not fulfill myeloma-defining criteria. Available clinical and laboratory evaluations did not reveal any SLiM-CRAB myeloma-defining events (9), including hypercalcemia, renal impairment, anemia attributable to plasma cell disorder, or bone lesions. Bone marrow examination showed a plasma cell burden of only 0.2% of nucleated cells, and the serum free light chain ratio was 0.71. These findings supported the diagnosis of MGUS without evidence of progression to symptomatic plasma cell myeloma.

### 2.3 Treatment and follow-up

The patient was diagnosed with DLBCL in the setting of hypertension and diabetes mellitus. Supportive treatment was initiated. Considering age and comorbidities, Pola-R-CHP was selected instead of R-CHOP according to CSCO guidelines (10). After the first cycle of Pola-R-CHP, the patient tolerated treatment well without severe hematologic or infectious complications. No treatment-related hospitalization occurred. Follow-up PET-CT and hematologic reassessment are planned after completion of subsequent treatment cycles.

## 3 Discussion

Previous studies have reported the coexistence of monoclonal gammopathy with various B-cell lymphoid malignancies, including gastrointestinal lymphomas. However, most reported cases describe monoclonal protein detection at or near the time of lymphoma diagnosis, whereas longitudinal evolution from long-standing immunoglobulin abnormalities to monoclonal gammopathy and subsequent lymphoma development remains rarely documented (11, 12). A central and distinctive feature of this case is the longitudinal evolution from long-standing polyclonal immunoglobulin elevation to the emergence of IgA-λ MGUS and subsequent development of colonic DLBCL (13), suggesting a possible multistep process of B-cell clonal evolution in the context of chronic immune perturbation.

The patient exhibited long-standing polyclonal immunoglobulin elevation accompanied by thyroid autoantibody positivity, suggesting a background of chronic immune activation (14). Notably, long-standing elevation of serum IgM was observed during disease evolution. Although the biological significance of this finding remains uncertain, sustained IgM elevation in the presence of autoimmune features may reflect prolonged antigenic stimulation and polyclonal B-cell activation. Chronic antigenic stimulation, autoimmune processes, and immune dysregulation have all been associated with sustained B-cell activation and increased risk of monoclonal gammopathy (8, 15). Prolonged immune stimulation may contribute to clonal selection and expansion of B cells, consistent with the later emergence of IgA-λ MGUS in this patient (16). However, this sequence should be interpreted as a temporal association rather than definitive clonal progression.

Importantly, age-related immune senescence and clonal hematopoiesis may have acted as biological contexts rather than direct causal drivers, facilitating genomic instability and altered immune surveillance. In the present case, the absence of clear light chain restriction in tumor tissue and lack of shared somatic mutations between plasma cells and lymphoma cells do not support a common clonal origin. Therefore, MGUS and DLBCL are more likely independent neoplastic processes arising within a shared immune-dysregulated background, rather than sequential stages of a single transformation pathway. This interpretation is consistent with emerging evidence that monoclonal gammopathies may coexist with or precede other lymphoid malignancies (17, 18).

Long-standing polyclonal hypergammaglobulinemia may reflect sustained immune activation and expansion of antigen-experienced B-cell populations (19). Over time, such a state may increase the probability of acquiring somatic or epigenetic alterations, thereby promoting clonal diversification under selective immune pressure. In elderly individuals, immune remodeling and clonal hematopoiesis may further impair immune surveillance and contribute to genomic instability (10). From this perspective, long-standing immunoglobulin abnormalities may reflect underlying immune system dysregulation in selected patients.

In addition to systemic immune factors, local microenvironmental influences may also contribute to lymphomagenesis. The development of primary intestinal DLBCL has been proposed to involve interactions among mucosal immunity, microbial antigens, and chronic inflammatory processes (20). However, the role of gut microbiota in this patient remains speculative, as no longitudinal microbiome analysis was performed.

NGS provided additional insight into the molecular background (21). *TET2* alterations have been associated with epigenetic dysregulation affecting hematopoietic differentiation and B-cell function (22). Recent studies have highlighted the potential clinical relevance of clonal hematopoiesis (CH) in patients with B-cell lymphomas. Beyond representing an age-associated accumulation of hematopoietic clones, CH may influence immune regulation and reshape the tumor microenvironment, thereby affecting lymphoma biology and clinical outcomes. In this context, CH may represent a potential biological background contributing to immune dysregulation in this patient (23). The *IDH2* alteration raises the possibility of altered cellular metabolism and epigenetic regulation through accumulation of oncometabolites (24). Notably, both *TET2* and *IDH2* alterations have been reported in age-related clonal hematopoiesis (25). However, because matched normal sequencing was not available, the presence of a CH-related background cannot be definitively established in this case.

Additionally, mutations in *IGLL5* and *DUSP2* may reflect abnormalities in B-cell receptor signaling and immune regulation, which have been reported in B-cell lymphomas (26). However, it is noteworthy that the IGLL5: c.72G>A (p.W24X) variant identified in this case showed a very low VAF of approximately 1%, suggesting that it may represent a subclonal event rather than a major clonal driver alteration. The transition from polyclonal immunoglobulin elevation to IgA-λ MGUS suggests emergence of a dominant plasma cell clone, potentially representing a key inflection point in B-cell clonal dynamics. However, the absence of immunoglobulin gene rearrangement analysis or BCR sequencing precludes confirmation of clonal continuity. Immunogenomic studies have shown that coexisting B-cell neoplasms may arise either from a common progenitor or independently under shared microenvironmental pressure (27, 28). Therefore, the relationship between MGUS and DLBCL in this case should be interpreted as hypothesis-generating rather than mechanistically proven.

Taken together, this case supports a conceptual framework in which chronic immune activation, age-related clonal hematopoiesis, and microenvironmental selection pressures collectively shape B-cell evolution. Rather than a linear transformation model, MGUS and DLBCL may represent temporally associated but manifestations arising within a shared dysregulated immune background. Patients with long-standing unexplained immunoglobulin abnormalities may benefit from longitudinal monitoring, as such findings could precede overt clonal hematologic disease (29). However, prospective studies are required before any surveillance recommendations can be established.

In terms of treatment, R-CHOP remains the standard first-line therapy for colonic DLBCL, although toxicity is a concern in elderly patients (30). The Pola-R-CHP regimen has shown favorable efficacy and safety and is increasingly considered an alternative in selected patients (31). In this case, the patient tolerated initial therapy well.

In summary, this case illustrates a possible multistep biological sequence linking chronic immune activation, emergence of monoclonal gammopathy, and lymphoma development. Rather than supporting a direct causal transformation, the findings favor a model of shared biological susceptibility and parallel clonal evolution. Further studies integrating longitudinal clinical observation with high-resolution molecular profiling are needed to clarify the relationship between long-standing immunoglobulin abnormalities, MGUS, and lymphoid malignancies.

## 4 Limitations

Several limitations should be acknowledged. First, clonality analysis, including immunoglobulin heavy-chain rearrangement or B-cell receptor sequencing, was not performed; therefore, a direct lineage relationship between MGUS and DLBCL could not be established. Second, matched normal sequencing, such as peripheral blood granulocyte sequencing, was not available; therefore, germline variants could not be completely excluded, and the distinction between tumor-associated alterations and age-related clonal hematopoiesis remains uncertain. Third, follow-up duration remains limited. Accordingly, the proposed biological relationship should be considered hypothesis-generating.

## 5 Conclusion

In summary, we report a rare clinical course characterized by long-standing polyclonal immunoglobulin elevation, subsequent emergence of IgA-λ MGUS, and later identification of primary colonic DLBCL. Rather than supporting a direct transformation from MGUS to lymphoma, the observed trajectory is more consistent with a shared biological background involving immune perturbation, age-related immune remodeling, and possible B-cell clonal selection. The coexistence of autoimmune features, evolving monoclonal gammopathy, and molecular alterations involving epigenetic regulators such as *TET2* and *IDH2* supports a possible multistep model of immune-associated clonal selection.

This case highlights that MGUS, in selected individuals, may occur within a broader context of immune dysregulation beyond plasma cell–restricted disorders. Although a direct clonal relationship between MGUS and DLBCL could not be established, the longitudinal immunological evolution observed in this patient emphasizes the potential value of individualized monitoring in patients with long-standing or evolving immunoglobulin abnormalities.

From a clinical perspective, patients with MGUS are generally monitored through periodic assessment of monoclonal protein levels, serum free light chains, complete blood counts, renal function, and calcium levels according to individual risk profiles. However, the significance of long-standing polyclonal immunoglobulin abnormalities preceding MGUS remains unclear. Evolving immunoglobulin patterns or additional immune abnormalities may warrant closer longitudinal evaluation. Although routine gastrointestinal endoscopic screening is not recommended for all MGUS patients, clinicians should remain attentive to gastrointestinal symptoms or other clinical indications requiring further evaluation.

## 6 Patient perspective

The patient stated that he had not previously considered the long-standing immunoglobulin abnormalities to be clinically significant because he had remained asymptomatic for many years. After being diagnosed with DLBCL, he expressed willingness to undergo regular follow-up and treatment. The patient tolerated the initial cycle of therapy well and hoped to maintain a stable quality of life during subsequent treatment.

## Data Availability

The original contributions presented in the study are included in the article/supplementary material. Further inquiries can be directed to the corresponding author.

## References

[1] Chowdhury SM Bhatta S Voorhees TJ Annunzio K Bond DA Sawalha Y . Impact of circulating lymphoma cells at diagnosis on outcomes in patients with newly diagnosed de novo diffuse large B-cell lymphoma. J Hematol Oncol. (2025) 18:4. doi: 10.1186/s13045-024-01658-y 39757235 PMC11702192

[2] Hasturk D Akturk Esen S Buyukaksoy M Civelek B Seven I Uncu D . Primary colon lymphomas: An analysis of our experience over the last 18 years. Med (Baltimore). (2024) 103:e38013. doi: 10.1097/md.0000000000038013 38728507 PMC11081556

[3] Hevroni G Vattigunta M Kazandjian D Coffey D Diamond B Maura F . From MGUS to multiple myeloma: Unraveling the unknown of precursor states. Blood Rev. (2024) 68:101242. doi: 10.1016/j.blre.2024.101242 39389906

[4] Quinn SJ Cardwell C Anderson L McShane C . A meta-analysis of the rate of Malignant progression of monoclonal gammopathy of undetermined significance: preliminary findings from a systematic review. HemaSphere. (2022) 6:1838–9. doi: 10.1097/01.hs9.0000850696.86402.84 33079766

[5] Trojani A Di Camillo B Bossi LE Greco A Leuzzi L Tedeschi A . Common gene expression signature of B-cells of Waldenström macroglobulinemia (WM) and IgM monoclonal gammopathies of undetermined significance (IgM MGUS) compared to healthy subjects. Blood. (2021) 138:4317–. doi: 10.1182/blood-2021-147712

[6] Jeryczynski G Krauth M-T . Monoclonal gammopathy of undetermined significance (MGUS): where is the hidden danger? Definition and work-up. memo - Magazine Eur Med Oncol. (2020) 14:76–9. doi: 10.1007/s12254-020-00630-z 30311153

[7] Sigurbergsdóttir A Love TJ Kristinsson SY . Autoimmunity, infections, and the risk of monoclonal gammopathy of undetermined significance. Front Immunol. (2022) 13:876271. doi: 10.3389/fimmu.2022.876271 35572590 PMC9096784

[8] Kallai A Ungvari Z Fekete M Maier AB Mikala G Andrikovics H . Genomic instability and genetic heterogeneity in aging: Insights from clonal hematopoiesis (CHIP), monoclonal gammopathy (MGUS), and monoclonal B-cell lymphocytosis (MBL). Geroscience. (2025) 47:703–20. doi: 10.1007/s11357-024-01374-y 39405013 PMC11872960

[9] Zanwar S Kumar S Rajkumar SV . Diagnosis, risk stratification and management of smouldering multiple myeloma. Nat Rev Clin Oncol. (2026) 23:279–96. doi: 10.1038/s41571-026-01119-0 41571958

[10] Zhao P Zhao S Huang C Li Y Wang J Xu J . Efficacy and safety of polatuzumab vedotin plus rituximab, cyclophosphamide, doxorubicin and prednisone for previously untreated diffuse large B-cell lymphoma: A real-world, multi-center, retrospective cohort study. Hematol Oncol. (2025) 43:e70017. doi: 10.1200/jco.2025.43.16_suppl.7061 39641321

[11] Boting W Weiguang W Feng L Shanhua Z Yunfeng C . IgA monoclonal gammopathy accompanying extranodal B cell lymphomas. Ann Hematol. (2014) 93:521–2. doi: 10.1007/s00277-013-1825-y 23793917

[12] Cox MC Esposito F Postorino M Venditti A Di Napoli A . Serum paraprotein is associated with adverse prognostic factors and outcome, across different subtypes of mature B-cell Malignancies-a systematic review. Cancers (Basel). (2023) 15:4440. doi: 10.3390/cancers15184440 37760410 PMC10527377

[13] Rajkumar SV Kyle RA Buadi FK . Advances in the diagnosis, classification, risk stratification, and management of monoclonal gammopathy of undetermined significance: Implications for recategorizing disease entities in the presence of evolving scientific evidence. Mayo Clin Proc. (2010) 85:945–8. doi: 10.4065/mcp.2010.0520 20884827 PMC2947967

[14] Smith MJ Rihanek M Coleman BM Gottlieb PA Sarapura VD Cambier JC . Activation of thyroid antigen-reactive B cells in recent onset autoimmune thyroid disease patients. J Autoimmun. (2018) 89:82–9. doi: 10.1016/j.jaut.2017.12.001 29233566 PMC5902436

[15] Dodig S Zrinski Topić R Demirović J Živčić J . Increased IgA in a subject with positive anti-thyroid autoantibodies - a case report. Biochem Med (Zagreb). (2010) 20:97–100. doi: 10.11613/bm.2010.012

[16] Liu Y Parks AL . Diagnosis and management of monoclonal gammopathy of undetermined significance: A review. JAMA Intern Med. (2025) 185:450–6. doi: 10.1001/jamainternmed.2024.8124 39960681 PMC11975479

[17] Kyle RA Larson DR Therneau TM Dispenzieri A Kumar S Cerhan JR . Long-term follow-up of monoclonal gammopathy of undetermined significance. N Engl J Med. (2018) 378:241–9. doi: 10.1056/nejmoa1709974 29342381 PMC5852672

[18] Lindqvist EK Lund SH Costello R Burton D Korde NS Mailankody S . Risk of progression in monoclonal gammopathy of undetermined significance (MGUS): Results from a population-based screening study. Blood. (2016) 128:5650–. doi: 10.1182/blood.v128.22.5650.5650

[19] Chen X Wang J Liu Y Lin S Shen J Yin Y . Primary intestinal diffuse large B-cell lymphoma: novel insights and clinical perception. Front Oncol. (2024) 14:1404298. doi: 10.3389/fonc.2024.1404298 39211552 PMC11357906

[20] Elkourashy SA Abu-El-Ruz R Askar MZ Hamdan A Zughaier SM . The microbiome-lymphoma Axis: A systematic review and Meta-analysis of gut Dysbiosis pattern in diffuse large B-cell lymphoma. Blood Rev. (2026) 75:101341. doi: 10.1016/j.blre.2025.101341 41238428

[21] Enssle JC Häupl B Qoku A Wang B Wright GW Barrans S . Pathogenesis and clinical relevance of diffuse large B-cell lymphoma proteogenotypes. Blood. (2024) 144:229–9. doi: 10.1182/blood-2024-204606

[22] Dominguez PM Ghamlouch H Rosikiewicz W Kumar P Béguelin W Fontán L . TET2 deficiency causes germinal center hyperplasia, impairs plasma cell differentiation, and promotes B-cell lymphomagenesis. Cancer Discov. (2018) 8:1632–53. doi: 10.1158/2159-8290.cd-18-0657 30274972 PMC6279514

[23] Maher N Moia R Almasri M Cividini L Genuardi E Cosentino C . Clonal hematopoiesis dynamics influences long-term outcomes of follicular lymphoma: Results from FIL FOLL12 trial. HemaSphere. (2026) 10:e70393. doi: 10.1002/hem3.70393 42256545 PMC13240525

[24] Ward JC Morgan M Wood J Woolley C de Menezes AAN Finch A . Analysis of IDH1 and IDH2 mutations as causes of the hypermethylator phenotype in colorectal cancer. J Pathol. (2025) 267:40–55. doi: 10.1002/path.6446 40545636 PMC12337813

[25] Dantec M Dufossé M Boudry A Fournier E Goursaud L Lebon D . Disease characteristics and monitoring of IDH1/IDH2-mutated acute myeloid leukemia. Blood Cancer J. (2025) 15:101. doi: 10.1038/s41408-025-01304-x 40410134 PMC12102383

[26] Li SS Zhai XH Liu HL Liu TZ Cao TY Chen DM . Whole-exome sequencing analysis identifies distinct mutational profile and novel prognostic biomarkers in primary gastrointestinal diffuse large B-cell lymphoma. Exp Hematol Oncol. (2022) 11:71. doi: 10.1186/s40164-022-00325-7 36243813 PMC9569083

[27] Kim D Park D . Deep sequencing of B cell receptor repertoire. BMB Rep. (2019) 52:540–7. doi: 10.5483/bmbrep.2019.52.9.192 31383253 PMC6774421

[28] Dunn-Walters D Townsend C Sinclair E Stewart A . Immunoglobulin gene analysis as a tool for investigating human immune responses. Immunol Rev. (2018) 284:132–47. doi: 10.1111/imr.12659 29944755 PMC6033188

[29] Alig SK Shahrokh Esfahani M Garofalo A Li MY Rossi C Flerlage T . Distinct Hodgkin lymphoma subtypes defined by noninvasive genomic profiling. Nature. (2024) 625:778–87. doi: 10.1038/s41586-023-06903-x 38081297 PMC11293530

[30] Lue JK O'Connor OA . A perspective on improving the R-CHOP regimen: From Mega-CHOP to ROBUST R-CHOP, the PHOENIX is yet to rise. Lancet Haematol. (2020) 7:e838–50. doi: 10.1016/s2352-3026(20)30222-2 33091357

[31] Thor D Cheekati M Brik B Polineni V Stack A . Real-world comparative outcomes of pola-R-CHP versus R-CHOP in patients with diffuse large B-cell lymphoma. Blood. (2025) 146:2759–. doi: 10.1182/blood-2025-2759

